# Consequences of “zombie-making” and generalist fungal pathogens on carpenter ant microbiota

**DOI:** 10.1016/j.cris.2024.100102

**Published:** 2024-11-30

**Authors:** Sophia Vermeulen, Anna M Forsman, Charissa de Bekker

**Affiliations:** a Department of Biology, University of Central Florida, Orlando FL 32816, USA; b Department of Biology, Colby University, Waterville ME 04901, USA; c Microbiology, Department of Biology, Utrecht University, 3584 CH Utrecht, the Netherlands

**Keywords:** *Ophiocordyceps*, *Beauveria*, Infection, Microbiome, Behavior manipulation

## Abstract

The bacterial microbiome of the ant *Camponotus floridanus* has been well characterized across body regions and maturation levels. However, potential effects of entomopathogens on the gut microbiome, and the fungal communities therein, are yet to be assessed. Additionally, the mycobiome remains often overlooked despite playing a vital role in gut ecology with potential implications for health and infection outcomes. We characterized the effects of two entomopathogens with different infection strategies on the gut micro- and mycobiota of *C. floridanus* over time; *Ophiocordyceps camponoti-floridani* and *Beauveria bassiana.* Specialist, ‘zombie-making’ *O. camponoti-floridani* fungi hijack the behavior of *C. floridanus* ants over three weeks, leading them to find an elevated position and fix themselves in place with their mandibles. This summiting behavior is adaptive to *Ophiocordyceps* as the ant transports the fungus to conditions that favor fruiting body development, spore production, dispersal, and transmission. In contrast, the generalist entomopathogen *B. bassiana* infects and kills the ant within a few days, without the induction of obvious fungus-adaptive behaviors. By comparing healthy ants with *Beauveria-* and *Ophiocordyceps-*infected ants we aimed to 1) describe the dynamics of the micro- and mycobiome of *C. floridanus* during infection*,* and 2) determine if the effects on gut microbiota are distinctive between fungi that have different infection strategies. While *Beauveria* did not measurably affect the ant host micro-and mycobiome, *Ophiocordyceps* did, especially for the mycobiome. Moreover, ants that were sampled during *Ophiocordyceps*-adaptive summiting behavior had a significantly different micro- and mycobiome composition compared to healthy controls and those sampled before and after manipulation took place. This suggests that the host microbiome might have a role to play in the manipulation strategy of *Ophiocordyceps*.

## Highlights

• *Ophiocordyceps* and *Beauveria* impact carpenter ant microbiota differently.
• The ant mycobiome is more diverse and dynamic during infection than the microbiome.
• Dysbiosis is significant in ants that display fungus-manipulated summiting behavior.
• *Ophiocordyceps* causes apparent dysbiosis while *Beauveria* infections do not.

## 1 Introduction

The genera *Beauveria* (family: Cordycipitaceae) and *Ophiocordyceps* (family: Ophiocordycipitaceae) represent well-studied examples of insect-infecting fungi (i.e., fungal entomopathogens) with different pathogenic strategies. *Beauveria bassiana* is a generalist that can infect insects across orders. As such, strains of this species are isolated and studied for their potential use in the biocontrol of insect pests (Maina et al., 2018, Mascarin and Jaronski, 2016). To initiate infection, *Beauveria* spores attach and penetrate through the insect host's cuticle. Once inside, the fungus proliferates as blastospores (i.e., single-celled, yeast-like cells), releases toxins, destroys host tissues, and kills the insect within a few days (Mascarin and Jaronski, 2016). This destructive, toxin-based infection strategy, paired with its wide host range classify *Beauveria* as a necrotroph-like fungal entomopathogen.

In contrast to *Beauveria, Ophiocordyceps* species are highly specialized entomopathogens that generally infect only one insect species (Araújo et al., 2015, Araújo et al., 2018, Araújo et al., 2020). The ‘zombie-making’ fungi of the species complex *Ophiocordyceps unilateralis* sensu lato infect ants from the Camponotini tribe. These fungi slowly hijack ant behavior over the course of three to four weeks before eventually killing their host (de Bekker et al., 2015, Will et al., 2020). While the fungus is growing as single blastospores, infected individuals wander away from the nest and climb to elevated positions that provide favorable conditions for fungal growth (Andersen et al., 2012, Will et al., 2022). The manipulated ant then proceeds to bite onto the vegetation, firmly attaching itself in the final moments before death. Following this summiting behavior, the fungus converts to a multicellular, mycelial growth, which eventually gives rise to a fruiting body that sprouts from the cadaver (Andersen et al., 2009, Fredericksen et al., 2017). This fruiting body carries the spores that are released to infect new ants (Araújo et al., 2015, Araújo et al., 2018, Araújo et al., 2020). These manipulated behaviors are a crucial aspect of the *Ophiocordyceps* life cycle that aid in circumventing social immunity behaviors of healthy nest mates (Trinh et al., 2021) and place the ant in a microclimate that promotes fungal growth and spore dispersal (Will et al., 2022, Andriolli et al., 2019). Without this behavior, the fruiting body cannot form (Andersen et al., 2009, Loreto et al., 2014), and transmission fails. Therefore, *Ophiocordyceps* species within the *unilateralis* complex must treat their host in a much more delicate manner than *B. bassiana* and use hemi-biotroph-like strategies (de Bekker et al., 2021) to ensure that the ant does not succumb to the infection prematurely. Comparable to hemibiotrophic plant pathogens, *Ophiocordyceps* begins its infection with a biotrophic-like phase, gaining resources from living cells by inducing subtle changes without causing severe tissue damage. Later in the infection, once manipulated biting occurs, the fungus switches to a necrotrophic lifestyle as it rapidly needs to consume insect tissue to gain enough energy for fruiting body development.

While there is previous work that connects insect pathologies (Boucias et al., 2018, Koch and Schmid-Hempel, 2012) and behavior (Jia et al., 2021, Teseo et al., 2019) with microbiota composition, the majority of microbiome research, including studies done on ants, focuses on comparisons between species, ecosystems, diet composition, and social status (Decker et al., 2023, Mendoza-Guido et al., 2023, Hu et al., 2018, Yun et al., 2018, Martins and Moreau, 2020, Ramalho et al., 2019, Ramalho and Moreau, 2023, Russell et al., 2009). Most of these studies have only focused on the bacterial microbiota, leaving the fungal microbiome (i.e., mycobiome) underexplored. Studies that have investigated insect mycobiomes suggest that fungal communities can also be impacted by disease and show different patterns in community composition when compared to the bacterial microbiome (Wu et al., 2020). Both the bacterial and fungal microbiome of grain beetles infected with tapeworms showed changes when compared to uninfected individuals (Fredensborg et al., 2020). Moreover, the gut mycobiome of honeybees was shown to be much more variable than their bacterial microbiome (Decker et al., 2023) and together with the bacterial gut community to be responsible for shaping social status (Yun et al., 2018). In fact, mycobiome explorations in mosquitoes demonstrated that interkingdom interactions between fungal and bacterial microbiota shape these communities, with consequences for host growth and development (Hegde et al., 2024). These studies suggest that research into insect mycobiomes would provide a more complete picture of the microbiome while potentially revealing a different side of microbiome-host interactions. Moreover, studies that investigate the effects of infectious diseases on the gut microbiota of vertebrates suggest their involvement in disease outcomes (Hahn et al., 2022, Dheilly et al., 2019, Haraoui and Blaser, 2023). With regards to the topic of fungal pathogens of insects, the intersection between insect pathology and the microbiome is even considered to be an avenue of research that may improve the use of these fungi in the biocontrol of insect pests (Boucias et al., 2018).

Currently, the interaction between the behavior-manipulating fungus *Ophiocordyceps camponoti-floridani* and its carpenter ant host *Camponotus floridanus* is one of the more in-depth studied examples of summit disease (Will et al., 2020, Will et al., 2022, Trinh et al., 2021, Das and de Bekker, 2022, Will et al., 2023, Will et al., 2023). Additionally, the bacterial microbiome of *C. floridanus* has been well characterized across different body regions (Ramalho et al., 2019) and maturation levels (Ramalho et al., 2017). Nevertheless, the fungal communities and effects from disease on *C. floridanus* microbiota have yet to be assessed. Since microbiome composition has been linked to behavior in other insects, and infection can seemingly change that composition, we propose that part of the behavioral changes observed in *Ophiocordyceps-*manipulated ants may be attributed to their microbiota. As such, we set out to investigate the bacterial and fungal gut microbiomes of *Ophiocordyceps*-infected *C. floridanus* using DNA metabarcoding. We compared our findings to data obtained from *Beauveria*-infected ants to answer if 1) fungal infection alters the bacterial and fungal community composition of the gut microbiome in *C. floridanus* when compared to healthy nestmates, 2) the gut microbiome community changes as disease progresses, and 3) the microbiome community is affected differently depending on the infecting entomopathogen (*Ophiocordyceps* vs *Beauveria*). We hypothesized that the microbiome of infected ants would diverge from that of healthy nestmates, and that *Ophiocordyceps* and *Beauveria* infections would affect microbiome compositions differently since they are fungi with contrasting parasitic lifestyles. If supported, this would suggest that changes in the host gut microbiota caused by *Ophiocordyceps* infection could underly some of the behavioral changes observed.

## 2 Methods

### 2.1 Ant collection and housing

Four *C. floridanus* colonies were collected in Seminole county, Florida: Two were collected from the University of Central Florida's (UCF) arboretum (28.6035, -81.1937) (Colony 1 and 2) and two colonies from the Chuluota Wilderness area (28.6214, -81.0556) (Colony 3 and 4). These collections were permitted by the UCF Arboretum and Seminole County Natural Lands, respectively. All collected colonies were queenless and averaged between one to two thousand individuals consisting of majors, minors, and brood.

Each colony was placed into a 9.4 L plastic container (Rubbermaid) with *ad libitum* water and food (i.e., frozen crickets and 15% sugar water). Several darkened 50 mL tubes (Greiner) served as nest chambers and talcum-lined walls prevented the ants from climbing out. To reset *C. floridanus* biological clocks to lab conditions, colonies were placed in an incubator (I36VL, Percival) under constant light, 25°C temperature, and 70% relative humidity for 48 hours. Subsequently, the colonies were entrained to 12 hr:12 hr light-dark cycles for three days at 28°C and 20°C, respectively, and 70% relative humidity. Each 24h cycle began with lights on at Zeitgeber time (ZT) 0, followed by a four-hour increasing ramping step to peak light settings and 28°C at ZT 4, a four-hour hold at peak light and temperature until ZT 8, and a four-hour decreasing ramp step to complete darkness and 20°C at ZT 12.

Seven days prior to *Beauveria* and *Ophiocordyceps* infections, two groups of 110 minor caste workers from each colony were separated into new 9.4 L plastic containers with a plaster bottom, which were kept under the same entrainment conditions. *Tillandsia* was draped on two upright 12 cm wooden skewers embedded in the plaster on one end of the container to provide the main biting substrate for *Ophiocordyceps*-infected *C. floridanus* in Central Florida (Will et al., 2022). On the other end of the container, a solid dark plastic bottom of a micropipette tip box (TipOne) was placed upside down to serve as a nest area. All fragment colonies were fed *ad libitum* on 15% sucrose solution and water throughout the experiment.

### 2.2 Fungal culturing and ant infections

*Ophiocordyceps* infections were performed with *O. camponoti-floridani* strain OJ 2.1 (Will et al., 2020). To obtain fresh *Ophiocordyceps* blastospore solution, 8 mL Grace's Insect Medium (Invitrogen) supplemented with 2.5% Fetal Bovine Serum (FBS) (Invitrogen) was inoculated with a frozen blastospore stock and incubated at 28°C in the dark at 50 rpm for seven days. *Beauveria* infections were done with *B. bassiana* strain ARSEF 2860 (USDA ARS Collection). To obtain fresh *Beauveria* blastospores solution, 20 mL Sabouraud dextrose broth (Sigma) was inoculated with fresh conidiospores and incubated at 25°C in the dark at 120 rpm. For six days. For both*Ophiocordyceps* and *Beauveria* infections, fungal blastospores were harvested immediately prior to injections by filtering the cultures through sterile gauze. Filtered blastospores were spun down and resuspended in fresh Grace's media supplemented with 2.5% FBS. *Ophiocordyceps* was resuspended to a concentration of 4.6 × 10^7^ blastospores/mL infection solution, while the *Beauveria* infection solution was brought to 3.5 × 10^7^ blastospores/mL.

Infections were performed with 10 µL borosilicate capillary tubes (Drummond) pulled to a needle point with a PC-100 Narishige instrument. To establish *Ophiocordyceps* infections, two times 110 ants, obtained from colony 1 and 2, were injected on the ventral side of the thorax under the first pair of legs with 0.5 uL of infection solution. The healthy control groups also consisted of two times 110 ants, taken from the same two colonies, sham-injected with 0.5 uL media without blastospores (Will et al., 2020). Following injection, the *Ophiocordyceps* treatment groups were left undisturbed for three days after which ants that died of injection trauma were removed. The 79% of *Ophiocordyceps*-injected and 85% of sham-injected ants that survived were kept in the infection experiment for further observation, data collection and sampling.

While injection is necessary for *Ophiocordyceps* infection, *Beauveria* infections are best established by pricking ants with a capillary needle that has been dipped into infection solution (Trinh et al., 2021). For *Beauveria* infections, two times 110 ants, obtained from colony 3 and 4, were infected by pricking them on the ventral side of the thorax under the first pair of legs with a blastospore-coated capillary needle. The healthy control groups of two times 110 ants, taken from the same colonies, were pricked with a needle dipped in media without fungal cells. The *Beauveria* treatment groups were left undisturbed for one day after which ants that died because of the procedure were removed. The experiment was continued with the 97% of *Beauveria*-pricked and 98% of sham-pricked ants that survived.

### 2.3 Daily observations, survival data collection and ant sampling

**Fig. 1:**
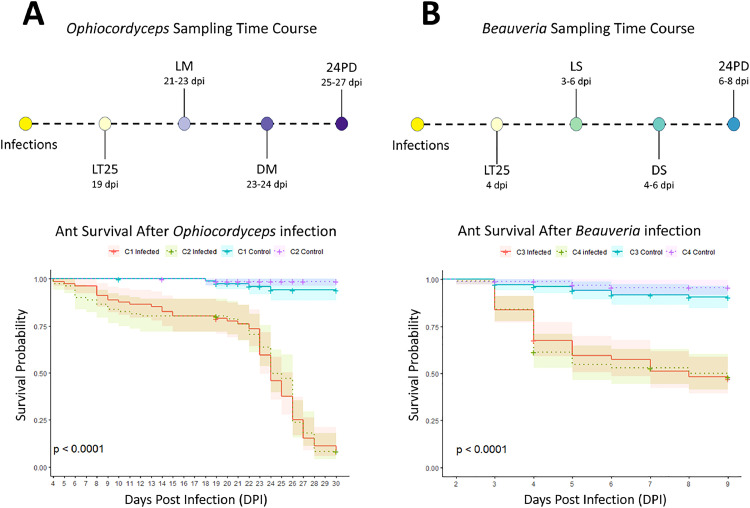
**Sampling regime and survival curves of C. floridanus ants infected with O. camponoti-floridani or B. bassiana.** Visualization of the parallel sampling timelines that were used to collect A) Ophiocordyceps-infected and B) Beauveria-infected ants for this study. The corresponding survival curves for both these infections are plotted underneath these timelines, demonstrating that both infections led to a significant reduction in survival probability for both infections as compared to healthy control ants obtained from the same colony (p < 0.0001). For both infections two independently collected ant colonies were used: colony 1 and 2 (C1 and C2) were used for Ophiocordyceps infections and colony 3 and 4 (C3 and C4) were used for infections with Beauveria. The two colony replicates for each infection showed a comparable survival probability. Samples were collected at the time when 25% of the ants had died (i.e., lethal time LT25), ants showed manipulated or sickness behavior (i.e., live manipulated LM and live sick LS), ants had died after showing these behaviors (i.e., dead manipulated DM and dead sick DS), and 24 hours after the ant had succumbed from the respective infections (i.e., 24 hours post death 24PD).

During infection, ten *Ophiocordyceps*-infected ants, ten *Beauveria*-infected ants, and ten sham-injected and -pricked control ants were collected at five successive timepoints: 1) prior to observation of infection-associated behaviors, 2) when infection-associated behaviors were observed and the ant was still alive, 3) when the ant had died after displaying infection-associated behaviors, and 4) 24h after the ant had died (Fig. 1). Details for each fungal infection type follow below. After sampling, we reduced the control group to comprise twelve healthy sham samples randomly selected from across the four *Ophiocordyceps-*infection timepoints and twelve from the *Beauveria*-infection timepoints. To maintain microbiome integrity, we flash froze ants in liquid nitrogen immediately following collection. Survival was recorded daily between ZT 5 and 7 (i.e., while lights were on) and dead individuals were removed.

At 19 days post infection (DPI) with *Ophiocordyceps*, 25% of the infected population had died, which we indicated as lethal time (LT) 25 (Fig. 1A). At this time, none of the infected individuals had shown manipulated summiting and biting behaviors yet, including those that had died. However, most individuals that make it across this time point eventually progress towards the ultimate summiting behavior (Will et al., 2020). As such, ten live ants were sampled at LT25 to investigate potential microbiome changes when infection had progressed but was still a few days prior to the manipulated summiting stage (Fig. 1A).

At 21 DPI, the first manipulated summiting and biting behaviors were observed in *Ophiocordyceps* infected individuals (Fig. 1A). This manipulation stage of the infection lasted until 28 DPI. During these days, manipulations started between ZT 18 and 22, but could be as late as ZT 1 in one case. To obtain live individuals that displayed the characteristic manipulated summiting and biting behavior (i.e., time point “Live Manipulation” or LM), ten ants were collected at ZT 22 to avoid disturbing others that were still in the process of climbing and biting. Live manipulated ants were still very responsive (i.e., moving legs and antennae rapidly when gently poked with forceps).

After manipulation, *Ophiocordyceps* is thought to rapidly switch its biotroph-like growth towards a more necrotrophic one as it begins to switch from single-celled blastospores to a multicellular hyphal growth, killing the ant host in the process (de Bekker et al., 2015). To capture potential microbiome changes during this process, ten ants were sampled that had just succumbed to the *Ophiocordyceps* infection after displaying manipulated summiting behavior (i.e., “Dead Manipulation” or DM) (Fig. 1A). Time of death was monitored hourly and occurred within a few hours upon manipulated biting. Ants were considered dead when they no longer moved in response to being poked with forceps.

Additionally, ten *Ophiocordyceps* infected ants were sampled 24h after manipulation and death when tissue damage due to cell death and fungal carbon and nutrient consumption had more time to progress (i.e., “24Hr Post Death” or 24PD (Fig. 1A). To collect these samples, dead manipulated individuals had to be moved to an isolated, identical container, kept within the same incubator to protect the dead ants from being removed/dismembered by their live nestmates.

To be able to compare *Ophiocordyceps* with *Beauveria* infection, ten *Beauveria-*infected and corresponding healthy controls were collected at comparable disease phenotypes (timepoints) throughout this infection. *Beauveria's* much more rapid disease progression required a different monitoring and sampling approach. Survival and behavioral observations were conducted every 4 hours. As infection neared the LT25 timepoint, observations were done every two hours to ensure precise timing. Because ant mortality at this point was a result from infection followed by rapid death, there was necessary overlap in the LT25 sample collection and later infection timepoints. The *Beauveria* infection reached LT25 at 4 DPI (Fig. 1B). To distinguish this first sampling group (LT25) from the second collection timepoint (infection-associated behavior), only ants that were out of the nest and able to walk at LT25 were collected.

Since *Beauveria* does not manipulate host behavior, for comparison ten *Beauveria-*infected ants were collected that displayed a distinct “sick” phenotype, starting at 3 DPI (i.e., “live sickness behavior” or LS) (Fig. 1B). Ants that displayed the “sick” phenotype could no longer walk more than a few steps without falling but still actively responded to a poke with forceps. They were often found on their sides while moving their legs but unable to stand. We considered this a comparable timepoint to the *Ophiocordyceps* ants collected at live manipulation because once *Beauveria-*infected ants displayed this behavior, they similarly died within a few hours (Fig. 1B).

To collect ten *Beauveria*-infected “dead” ants, “sick” ants were isolated in identical container setups to prevent disturbance from nest mates and monitored hourly until death, after which they were immediately sampled (i.e., “dead sickness behavior”, or DS). To collect *Beauveria*-infected ants 24h post death (24PD), dead ants that had displayed the “sick” phenotype were placed in isolation for 24h under the same incubator conditions as the remainder of the experiment (Fig. 1B).

### 2.4 DNA extraction, library preparation, and sequencing

All samples were processed in a randomized order for bacterial 16S and fungal ITS metabarcoding (Supplementary File 1, see metadata sheets). Gasters were separated from frozen ants under sterile conditions and placed in 2 mL Eppendorf tubes with two metal ball bearings (5/32” type 2B, grade 300, Wheels Manufacturing) to disrupt cells in a Mini G Tissue Homogenizer (SPEX) for 60 seconds at max rpm. Negative DNA extraction controls were also included. DNA was extracted from these samples using the ZymoBIOMICS DNA microprep kit (Zymo Research, D4301) following manufacturer's recommendations. Amplicon libraries were prepared according to the 16S Metagenomic Sequencing Library Preparation protocol from Illumina.

For bacterial metabarcoding, the V4 region of the 16S SSU rRNA gene was targeted using primers 515F (Parada et al., 2016) and 806R (Apprill et al., 2015). For fungal metabarcoding, the internal transcribed spacer ITS2 region was targeted using ITS primers ITS3_KYO2 (Toju et al., 2012) and ITS4 (White et al., 1990). Amplicon PCR was conducted in 25 µL reactions using 2.5 µL of DNA sample, 5 µL each of 1 µM forward and reverse primer, and 12.5 µL of KAPA HiFi HotStart ReadyMix (Roche). The 16S amplicon reactions were performed in duplicate using the following PCR conditions: initial denaturation at 95°C for 3 minutes, followed by 25 cycles of 95°C for 30 seconds, 50°C for 30 seconds and 72°C for 30 seconds, and a final extension at 72°C for 5 minutes. The ITS reactions were performed in singleton using the same PCR conditions, but the annealing temperature used was 47°C. Negative PCR controls were included in these reactions. Clean-up of the PCR products was done with 1.5x Sera-Mag SpeedBead solution following the protocol described in (Forsman et al., 2021). The duplicate 16S reactions were combined during this process. The fragment size of randomly selected 16S and ITS libraries was checked using a Tapestation 4200 (Agilent) and D1000 HS reagents (Agilent). For both 16S and ITS, we detected expected fragment sizes between 300-400 bp.

We uniquely dual-indexed all ITS libraries in 50 µL PCR reactions, consisting of 8 µL PCR amplicon, 5 µL each of Nextera XT Index primers i5 and i7 (Illumina), 7 µL molecular grade water (Invitrogen), and 25 µL KAPA HiFi HotStart ReadyMix (Roche). The 16S libraries were indexed in 25 µL PCR reactions consisting of 8.5 µL PCR amplicon, 2 µL each of Nextera XT Index primers i5 and i7, and 12.5 µL KAPA HiFi HotStart ReadyMix. The PCR conditions for both indexing reactions were as follows: initial denaturation at 95°C for 3 minutes, followed by 8 cycles of 95°C for 30 seconds, 55°C for 30 seconds and 72°C for 30 seconds, and a final extension at 72°C for 5 minutes. Indexed PCR products were again cleaned with 1.2x Sera-Mag SpeedBead solution.

Following indexing, all libraries were quantified using a Qubit fluorometer with the 1X dsDNA HS assay kit (Invitrogen) to create an equimolar pool for sequencing. After an additional 1.2x magnetic bead cleanup, a high and a low concentration pool were created and quantified a NEBNext Library Quant Kit for Illumina (New England Biolabs). Average fragment size and adaptor dimer removal were determined on a Tapestation 4200 (Agilent) with D1000 HS reagents (Agilent). Finally, the 16S and ITS library pools were combined in equimolar amounts for sequencing using a paired-end approach (2 × 300bp) and V3 reagents (Illumina) on an Illumina MiSeq instrument.

### 2.5 Sequence data processing

Paired-end sample reads were demuliplexed on the MiSeq instrument prior to importing data into QIIME2 (Bolyen et al., 2019) for further processing and quality control (see Supplementary File 2 for all code used). To produce amplicon sequence variants (ASVs), reads were trimmed with QIIME2 *cutadapt trim-paired* and denoised, dereplicated, and merged with *dada2 denoise-paired*. For ITS reads, denoising was performed with and without truncation (QC or otherwise) to determine the most optimal procedure for our data. Though it is generally not recommended to trim reads at the 3’ end to prevent bias in amplicon size (Rolling et al., 2022), truncation *(trunc-len-f 276; trunc-len-r 173)* proved to have the most classified reads compared to the other methods. Therefore, we used truncated data for downstream analyses while acknowledging that doing so may have introduced some bias in amplicon size.

Taxonomic classification of 16S ASV's was done using the Green Genes 13.5 database at 99% identity with BLAST+ (QIIME2, *classify-consensus-blast*; (Camacho et al., 2009)). A phylogenetic reference tree was created with a Green Genes 13.8 SATé-enabled phylogenetic placement database (QIIME2, *fragment-insertion sepp*; (Janssen et al., 2018). Classification of ITS ASV's was done using a naïve bayes classifier (QIIME2, *fit-classifier-naive-bayes*) trained on the fungal 9.0 UNITE database (Abarenkov et al., 2023). After assigning taxonomy, 16S reads not identified to at least phylum, assigned being of chloroplast or mitochondrial origin, and those that did not meet the set minimum of three reads per ASV were filtered out. Rarefaction curves (package: vegan – *rarecurve*) indicated that a sequencing depth of 4000 was sufficient to capture most ASVs. Of all our collected samples, 94 passed this cutoff (negative extraction and PCR controls not included) (Supplementary File 3, Fig. 1 and Table 1). Similarly to 16S filtering, ITS reads were excluded that were not identified to the phylum-level and below the required minimum of three reads per ASV. We also filtered out reads identified as *Ophiocordyceps spp.* and *Beauveria spp.* as these were the infecting entomopathogens. Rarefaction curves indicated sufficient ASV coverage at a sequencing depth of 500. Out of all our samples, 81 samples passed this cutoff (Supplementary File 3, Fig. 1 and Table 1).

### 2.6 Statistical analyses

All statistical analyses were conducted in R version 4.2.1 (see Supplementary File 2 for all code used). Survival probability of ants across the infection period was analyzed with the R package survival version 3.5-8 (Therneau, 2024) and survival curves were constructed using survminer version 0.4.9 (Kassambara et al., 2021).

To determine alpha and beta diversity classified reads and taxonomy data were paired with sample metadata and imported into R as phyloseq objects. Within-sample alpha diversity was calculated using observed richness and Shannon diversity (package: phyloseq – *estimate_richness*) (McMurdie and phyloseq, 2013). To test for differences between diversities, a Kruskal-Wallis rank sum test was used, followed by pairwise comparisons using the Dunn test (package: dunn.test - *dunn.test*, p-value adjustment method for multiple comparisons = Bonferroni) (Dinno, 2024). Alpha diversity differences were visualized with box plots using ggplot2 (Wickham, 2016).

Differences between groups and timepoints were also investigated using beta diversity metrics and pairwise permutational multivariate analyses of variance (PERMANOVA) (package: pairwiseAdonis – *pairwise.adonis,* method = bray curtis, permutations = 999, p-value adjustment method for multiple comparisons = Bonferroni) (Martinez Arbizu, 2017). Beta diversity was visualized using non-metric multidimensional scaling (NMDS) ordination of Bray-Curtis distances (package: phyloseq – *plot_ordination*) (McMurdie and phyloseq, 2013) and taxa bar plots were produced using ggplot2 (Wickham, 2016).

Using these same methods to detect differences in alpha and beta diversity, we compared healthy control ants that were obtained from the same colonies as the *Ophiocordyceps* infection (Colony 1 and 2) and the *Beauveria* infection (Colony 3 and 4) and found that their microbiota had no significant differences in alpha or beta diversity. This indicated that the ants used in these infection experiments had similar microbiota despite being from different colonies and field collections, allowing for direct comparisons between the two fungal infection treatments. Therefore, we combined them into a single healthy control group for comparisons to the infected ant groups.

To determine whether the type of fungal infection (i.e., necrotroph-like versus hemi-biotroph-like) affected the bacterial and fungal communities of the gut microbiome differently, reads from timepoints LT25 through 24HR were grouped into two categories based on the infecting entomopathogen (treatment groups): *Ophiocordyceps-* or *Beauveria-*infected ants. For timepoint comparisons, reads from the two fungal groups were separated back into their respective timepoints: control, LT25, LM/LS, DM/DS, 24PD. In doing so, we discovered one outlier ITS *Beauveria* 24HR sample that had high NMDS1 and NMDS2 scores (Supplementary File 3, Fig. 2). After the filtering steps, and removal of *Ophiocordyceps* and *Beauveria* reads, this sample was only left with unidentified reads and reads identified as *Malassezia*, a likely contaminant. Therefore, we removed this sample from both alpha and beta diversity analyses.

**Fig. 2:**
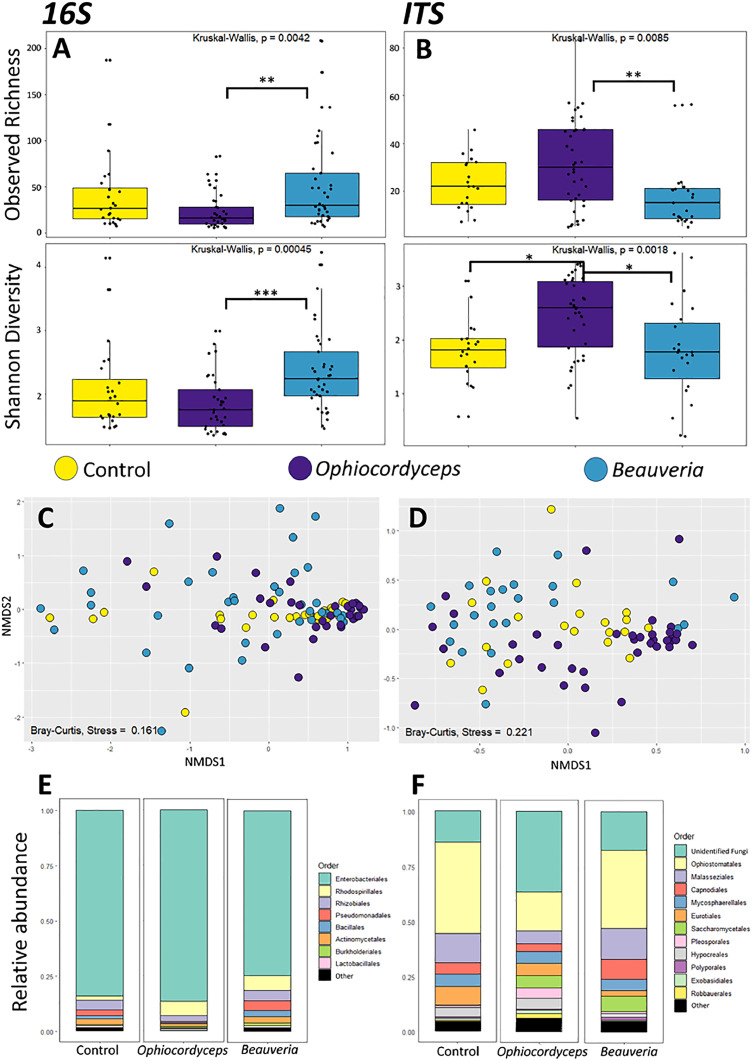
**Differences in bacterial microbiota and fungal mycobiota between healthy C. floridanus ants and those infected with O. camponoti-floridani or B. bassiana**. Box plot representations of the bacterial (16S) (A) and fungal (ITS) (B) alpha diversity in the guts of healthy control ants, ants infected with Ophiocordyceps, and ants infected with Beauveria. Adjusted p-values of significantly different observed ASV richness and Shannon diversity between infection groups are indicated as follows: * p<0.05, ** p< 0.01, *** p<0.001. (C and D) Beta diversity is depicted in non-metric multidimensional scaling (NMDS) plots for the different infection treatment groups. The points represent bacterial (D) and fungal (E) communities from individual gut samples, and their color indicates which treatment group each sample belongs to. C) NMDS plot using a Bray Curtis distance matrix of the bacterial gut microbiome for different infection groups (stress = 0.161). D) NMDS plot using a Bray Curtis distance matrix of the fungal gut mycobiome for different infection groups (stress = 0.22). E and F) Taxa bar plots showing the relative abundance of bacterial (E) and fungal (F) orders present in the guts of healthy control ants, ants infected with Beauveria, and ants infected with Ophiocordyceps.

## 3 Results

### 3.1 Overall data quantity and quality

Sequencing resulted in 6,670,956 paired-end reads across all samples for 16S libraries and 5,968,530 paired-end reads for ITS libraries. After filtering, taxonomic assignment, and rarefaction, 1280 ASVs were retained across 94 samples for 16S libraries and 833 ASVs across 81 samples for ITS libraries (Supplementary File 3, Table 1).

To monitor and account for contamination introduced during DNA extraction and library preparation, negative controls for DNA extraction and library preparation were included. Both negative controls had a read coverage similar to biological samples and comparable numbers of detected taxa. Consequently, we could not reliably use these controls to remove potential contaminants from our samples. To investigate the source of contaminant introduction, samples were grouped by DNA extraction date and PCR date and alpha diversity metrics were analyzed. While certain extraction dates were significantly different, there was no gradual increase in richness or Shannon diversity over time (Supplementary File 3, Fig. 3). This indicates that the reagents themselves were not contaminated during the process and that the taxa observed in the negative controls were likely introduced due to some level of cross-contamination during extraction. Grouping samples by PCR date did not show any significant differences (Supplementary File 3, Fig. 4), indicating that no measurable additional cross-contamination was introduced during library preparation. Taken together, our biological samples have some level of cross-contamination that might obscure present differences between groups. However, because we processed all samples in a random order (both extractions and PCR reactions), any significant differences detected between treatment groups or infection time points cannot be attributed to batch effects from the days they were processed.

**Fig. 3:**
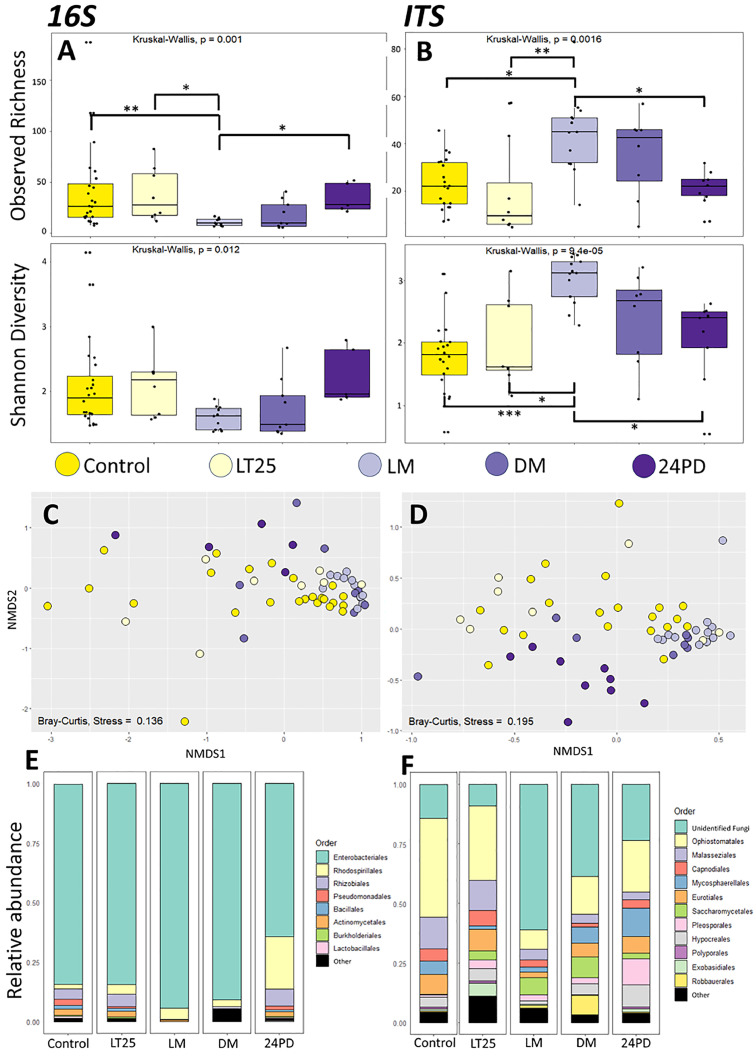
**Differences in bacterial and fungal gut microbiota of C. floridanus during infection progression and manipulation by O. camponoti-floridani**. Box plot representations of bacterial (16S) (A) and fungal (ITS) (B) alpha diversities in the guts of healthy control ants and ants infected with Ophiocordyceps, which were sampled before (LT25), during (LM) and after manipulated summiting and biting behavior (DM and 24PD). Adjusted p-values of significantly different observed ASV richness and Shannon diversity between infection groups are indicated as follows: * p<0.05, ** p< 0.01, *** p<0.001. C and D) Beta diversity is depicted in non-metric multidimensional scaling (NMDS) plots for the different Ophiocordyceps infection time points. The points represent bacterial (D) and fungal (E) communities from individual gut samples, and their color indicates which infection time point each sample belongs to. C) NMDS plot using a Bray Curtis distance matrix of the bacterial (16S) gut microbiome for different infection time points (stress = 0.135). D) NMDS plot using a Bray Curtis distance matrix of the fungal (ITS) gut mycobiome for different infection time points (stress = 0.195). E and F) Taxa bar plots at order level showing the relative abundance of bacterial (E) and fungal (F) microbiota present in the guts of healthy control ants, and ants infected with Ophiocordyceps prior (LT25), during (LM) and after manipulation (DM and 24PD).

**Fig. 4:**
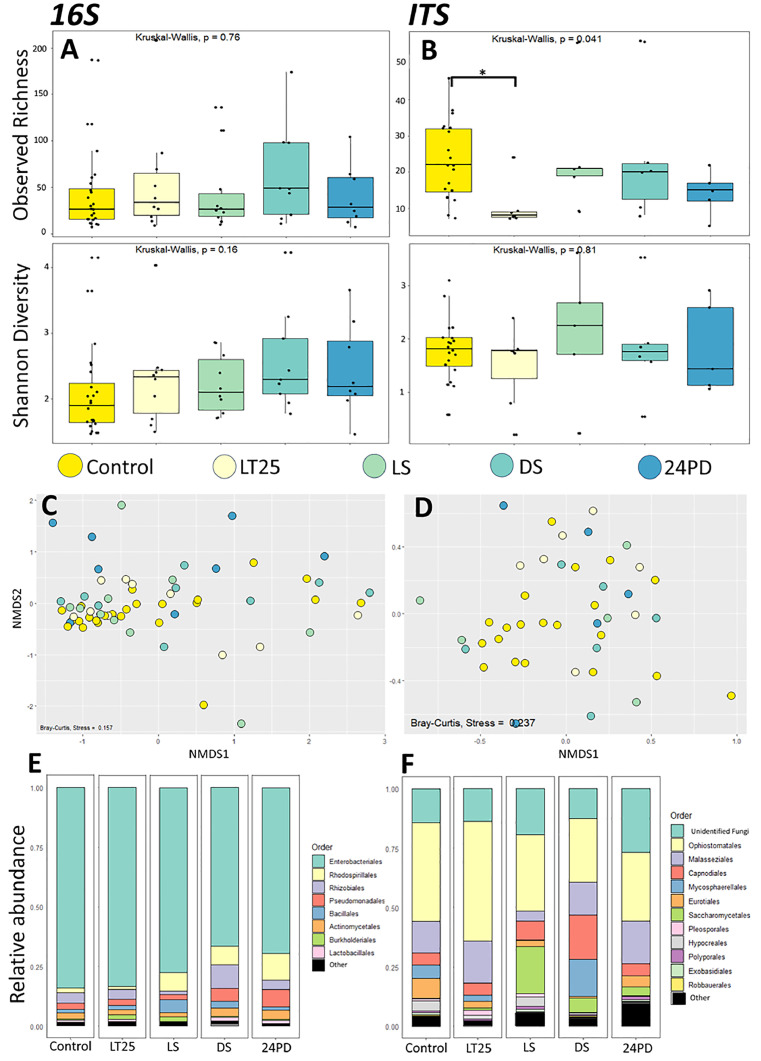
**Bacterial and fungal gut microbiota of C. floridanus during infection by B. bassiana**. A and B) Box plot representations of the bacterial (16S) (A) and fungal (ITS) (B) alpha diversity within the guts of healthy control ants and ants infected with Beauveria, which were sampled at different time points during infection. The adjusted p-value of the sole significantly different observed ASV richness between 24 hour after death (24PD) and healthy control is indicated as * p<0.05. C and D) Beta diversity is depicted in non-metric multidimensional scaling (NMDS) plots for the different Beauveria infection time points. The points represent bacterial (D) and fungal (E) communities from individual gut samples, and their color indicates which infection time point each sample belongs to. C) NMDS plot using a Bray-Curtis distance matrix of the bacterial (16S) gut microbiome for different infection time points (stress = 0.159). D) NMDS plot using a Bray-Curtis distance matrix of the fungal (ITS) gut mycobiome for different infection time points (stress = 0.236). E and F) Taxa bar plots at order level showing the relative abundances of bacterial (E) and fungal (F) taxa present in the guts of healthy control ants, and ants infected with Beauveria prior to displaying a “sick” phenotype (LT25), during (LS) and after displaying the “sick” phenotype (DS and 24PD).

### 3.2 Differences in alpha diversity between infection treatments

When the alpha diversity of gut microbiota in healthy ants and those infected with *Ophiocordyceps* and *Beauveria* were compared, no effect of fungal infection on bacterial (16S) alpha diversity was found (Fig. 2A). However, the mycobiome (ITS) in *Ophiocordyceps-*infected ants had a significantly higher Shannon diversity index (p = 0.01) than healthy control ants (Fig. 2B, see Supplementary File 4, Table 2 for all test and p-values). These results indicate that *Ophiocordyceps* infection may affect the relative abundance of fungal taxa present within the healthy ant host gut.

When the effects of *Ophiocordyceps* and *Beauveria* infections were compared to one another, the alpha diversities of both the bacterial microbiome and fungal mycobiome were found to be significantly different (Fig. 2A and B). The bacterial gut microbiome of *Ophiocordyceps*-infected ants had a significantly lower observed ASV richness (p=0.003) and Shannon diversity index (p = 0.0003) than those infected with *Beauveria* (Fig. 2A, Supplementary File 4, Table 2). The reverse was observed for the fungal gut mycobiome; the mycobiome of ants infected with *Ophiocordyceps* had a significantly higher observed richness (p = 0.006) and Shannon diversity index (p = 0.01) than the mycobiome of ants infected with *Beauveria* (Fig. 2B, Supplementary File 4, Table 2). This suggests that these two fungal pathogens with different infection strategies, affect the host microbiome and mycobiome differently.

### 3.3 Beta diversity between infection treatments

Beta diversity metrics were also used to determine if there were differences in gut micro/mycobiome community compositions between healthy and fungal-infected ants. These metrics corroborated our alpha diversity results. The bacterial community compositions were again found to not be significantly different between infected ants and healthy controls (Fig. 2C). However, the fungal community compositions were again significantly different between the *Ophiocordyceps* infected ants and healthy controls (PERMANOVA, p = 0.003) (Fig. 2D, Supplementary File 4, Table 3). Moreover, the beta diversity metrics also indicated that both bacterial and fungal community composition differed significantly between ants in the *Ophiocordyceps* and *Beauveria* treatment groups (PERMANOVA, p = 0.033 and p = 0.003, respectively) (Fig. 2C and D, Supplementary File 4, Table 3).

In line with findings from previous microbiome work done on *C. floridanus* (Ramalho et al., 2019, MO et al., 2017), Enterobacteriaceae (Order Enterobacteriales) constituted the most abundant family of bacteria in the gut microbiome of ants from all treatment groups and were predominately composed of Genus *Blochmannia,* a known endosymbiont of the C*amponotini* tribe. When inspecting relative taxa abundance for the bacterial microbiome (Fig. 2E, Supplementary File 1), a decrease in the relative abundance of Order Enterobacteriales in *Beauveria* infected ants compared to *Ophiocordyceps*-infected ants was observed. This might be the underlying cause of the significant difference between the bacterial gut microbiota of *Ophiocordyceps-* and *Beauveria-*infected ants. Determining relative taxa abundance for the gut mycobiome (Fig. 2F) it was found that *Ophiocordyceps*-infected ants contained a greater percentage of unclassified fungal taxa in their guts compared to the *Beauveria*-infected and healthy control ants, which likely accounts for the found difference in community composition. *Beauveria-*infected and control ants also contained a greater percentage of fungi within Order *Ophiostomatales*, mainly comprised of the species *Sporothrix insectorum,* which is regularly reported to be a fungal pathogen of insects (Li et al., 2010, Luz et al., 2007)*.*

### 3.4 Differences in alpha diversity in *Ophiocordyceps*-infected ants over time

The alpha diversities of gut microbiota and mycobiota were also compared between healthy ants and infected ants at four, progressive time points following *Ophiocordyceps* inoculation. These time points comprised 1) LT25 (lethal time 25%): infected, live ants, before the ultimate manipulated summiting and biting phase; while 25% had succumbed to the infection, 2) LM (live manipulation): live ants that displayed manipulated summiting and biting behavior, 3) DM (dead after manipulation): ants that died a few hours after manipulation took place, and 4) 24PD (24hr post death after manipulation): ants that had been dead for 24 hours after manipulation (Fig. 3A and B). The ASV richness of the bacterial microbiome of LM ants was observed to be significantly lower than for healthy controls (p = 0.005), live ants prior to manipulation (LT25, p = 0.022), and those 24 hours after death (24PD, p = 0.03) (Fig. 3A) (Supplementary File 4, Table 4). Only the dead manipulated time point (DM), which was sampled only a few hours later, was found to be similar in ASV richness to LM. The mycobiome was found to have the opposite pattern (Fig. 3B). Live manipulated (LM) ants were observed to have higher fungal ASV richness in their guts than healthy control ants (p = 0.014), infected ants before manipulation (LT25, p = 0.006), and those sampled 24 hours after dying in the manipulated summiting and biting position (24PD, p = 0.048) (Supplementary File 4, Table 4). Alpha diversity differences in the mycobiome among time points were again more apparent than alpha diversity differences in the bacterial microbiome. The same significant differences in Shannon diversity index were found across timepoints in *Ophiocordyceps-*infected ants (Fig. 3B); LM samples had a higher Shannon diversity index compared to healthy control samples (p < 0.0001), LT25 (p = 0.012), and 24PD samples (p = 0.034) (Supplementary File 4, Table 4). Taken together, these results suggest that the gut micro- and mycobiome of ants that show *Ophiocordyceps*-adaptive summiting and biting behaviors are significantly different compared to healthy ants, and even those in other stages of the infection.

### 3.5 Beta diversity between *Ophiocordyceps*-infected ants over time

Weighted beta diversity metric (Bray-Curtis) and relative abundances of community constituents were also used to investigate potential differences in gut microbiome and mycobiome composition between behaviorally manipulated ants (LM) and other stages of infection. For the bacterial microbiome of *Ophiocordyceps-*infected ants (Fig. 3C), only significant differences in community composition between the guts of live manipulated ants (LM) and those that were sampled 24 hours after manipulation (24PD) and death (DM) were found (PERMANOVA, p = 0.04) (Supplementary File 4, Table 5). In contrast, the mycobiome of *Ophiocordyceps-*infected ants (Fig. 3D) was significantly different in community composition compared to several timepoints. The gut mycobiome of ants that were sampled during manipulated summiting behavior (LM) were found to have significantly different fungal community composition compared to healthy control ants (p = 0.01), infected ants prior to manipulation (LT25, p = 0.02) and ants that were sampled 24 hours after they succumbed to the infection (24PD, p = 0.01) (Supplementary File 4, Table 5). These results support the evidence from the alpha diversity analyses that the fungal mycobiome is significantly different in *Ophiocordyceps*-infected ants during manipulated summiting and biting behavior as compared to healthy ants and other infection time points. A sudden increase in the relative abundance of unclassified taxa at the behavioral manipulation (LM) timepoint and a large decrease in the relative abundance of the most prevalent Order *Ophiostomatales* was observed (Fig. 3F, Supplementary File 1). In the two following time points sampled after behavioral manipulation (DM and 24PD), again an increase in the relative abundance of *Ophiostomatales* and a decrease in unclassified taxa was found, indicating a gradual shift in community composition.

### 3.6 Alpha and beta diversity in *Beauveria*-infected ants over time

We also looked into the gut micro- and mycobiota of *Beauveria-*infected ants at time points that parallel those investigated for *Ophiocordyceps* infection. As such, *Beauveria*-infected ants were sampled at LT25, when showing non-manipulative “sick behavior” (LS), having died after showing sick behavior (DS) and 24 hours post death (24PD). While the bacterial and fungal gut alpha diversities of *Ophiocordyceps*-infected ants that showed the manipulation phenotype (LM) were found to be different from healthy and other infection stages (Fig. 3), the gut compositions of *Beauveria*-infected ants that showed the paralleling “sick” phenotype (LS) were not (Fig. 4). Overall, no significant differences in the observed richness and Shannon diversity of the bacterial microbiome were found (Fig. 4A, Supplementary File 4, Table 6). Only a significant difference in the observed richness of the fungal mycobiome between healthy control ants and *Beauveria*-infected ants that had not yet shown the sick phenotype was detected (LT25, p = 0.034) (Supplementary File 4, Table 6). However, the results for Shannon diversity analyses did not corroborate this finding (Fig. 4B). Using the Bray-Curtis metric for beta diversity yet another, singular and distinct result was found; the bacterial microbiome composition at the 24-hour postmortem time point (24PD) was found to be significantly different compared to healthy control ants (PERMANOVA, p=0.01). The bacterial microbiome composition also differed significantly between the early *Beauveria*-infection time point in which a sick phenotype was not detected yet (LT25) and the 24PD time point (PERMANOVA, p = 0.04) (Fig. 4C, Supplementary File 4, Table 7). For the fungal mycobiome none of the time point comparisons were found to be significantly different in their composition (Fig. 4D, Supplementary File 4, Table 7). These results suggest that *Beauveria* infection does not affect the host bacterial microbiome or fungal mycobiome consistently enough to detect significant differences between infection stages. Indeed, when investigating the relative abundance of bacterial and fungal taxa across *Beauveria* infection time points, no large changes in taxa abundance were observed (Fig. 4E and F). Moreover, the mycobiome of *Beauveria-*infected ants (Fig. 4F) was dominated by *Ophiostomatales* while the relative abundance of unidentified fungal taxa was a lot smaller. This is in stark contrast with the relative abundances of fungal gut taxa that were observed in the guts of *Ophiocordyceps*-infected ants (Fig. 3F). There, a relative increase of unidentified fungal gut taxa was found, while the abundance of *Ophiostomatales* decreased. The increased relative abundance of these unidentified fungal gut taxa, especially during behavioral manipulation, might be a specific hallmark of the infection strategies of *Ophiocordyceps*.

## 4 Discussion

We investigated the effects of infection with *O. camponoti-floridani* and *B. bassiana* on the gut microbiota and mycobiota of the carpenter ant *C. floridanus. Ophiocordyceps camponoti-floridani* is a specialist, behavior-manipulating, hemi-biotroph-like pathogen of *C. floridanus*, and *B. bassiana* is a generalist, non-manipulating, necrotroph-like pathogen. When comparing the bacterial microbiome diversity (16S) of healthy ants to their *Ophiocordyceps*- and *Beauveria*-infected counterparts we did not find any significant differences. The bacterial community composition of infected ants remained relatively similar to that of controls. This would indicate that fungal infection is not impacting the ant gut microbiome as hypothesized. However, we found that the fungal mycobiome (ITS) of *Ophiocordyceps-*infected ants had a significantly higher ASV diversity (Shannon index) than the mycobiome of control ants with corroborating differences in community composition. Thus, based on the results we observed for gut mycobiota, we conclude that behavior-manipulating *Ophiocordyceps* infection does, in fact, affect the host gut. During *Ophiocordyceps* infection, the fungal mycobiome appears to be more dynamic in its composition as compared to the bacterial microbiome, with relative abundances of fungal gut taxa changing upon infection. *Blochmannia* was by far the most dominant bacterial genus within the carpenter ant microbiome, both in this and other studies (Ramalho et al., 2019, MO et al., 2017), which may have obscured changes other than those in the dominating species. Instead, there may be higher evenness between fungal taxa than between bacterial taxa, making subtle differences in the mycobiome easier to detect. Studies in other insect species also suggest that the fungal mycobiome may be more variable than the bacterial microbiome (Decker et al., 2023, Višňovská et al., 2020). Moreover, we detected a dominant presence of *S. insectorum*, which is commonly known as an entomopathogen (Li et al., 2010, Luz et al., 2007), but not necessarily as an insect gut symbiont. In line with this, other known entomopathogens, of the genus *Ophiocordyceps,* have been detected in the mycobiomes of plants and beetles (Matsuura et al., 2018, Zhong et al., 2014). As such, exploring the mycobiome can also reveal alternative lifestyles of presumed primary pathogens. Moreover, studies in humans and other animals (including disease vectors such as mosquitoes (Angleró-Rodríguez et al., 2016)) clearly indicate that the fungal mycobiome influences disease susceptibility and progression (reviewed in (Zhang et al., 2021, El-Jurdi and Ghannoum, 2017)). Taken together, this all suggests that it is worthwhile to include the mycobiome in insect microbiota studies, which are currently still largely overlooked.

We also found that both bacterial and fungal diversity and composition of the gut community were significantly different between ants infected with *Ophiocordyceps* and *Beauveria*. The bacterial microbiome of *Ophiocordyceps*-infected ants had a significantly lower ASV diversity than the microbiome of the *Beauveria*-infected ants. In contrast, the mycobiome of *Ophiocordyceps*-infected ants had a significantly higher ASV diversity than in *Beauveria*-infected ants. These results indicate that these entomopathogenic fungi, with contrasting infection strategies, impact the overall gut microbiota differently. Additionally, the specialist, behavior-manipulating fungus *Ophiocordyceps* caused a significant dysbiosis while the generalist fungus *Beauveria* did not seem to affect the gut composition much. Indeed, when we compared control ants to those at progressive infection time points, we did not detect consistent, significant changes in the gut communities of *Beauveria*-infected ants when analyzing alpha and beta diversity. This suggests that *Beauveria* infection leaves the gut composition relatively unchanged, with perhaps some gradual changes leading up to the fungus’ saprophytic growth after death as the significant differences in beta diversity between the 24 hours post death (24PD) time point and the healthy and early infection time point (LT25) suggest.

In contrast, in *Ophiocordyceps*-infected ants, the micro- and mycobiome both displayed a notable shift in ASV richness at live manipulation (LM) relative to both earlier (i.e., control and LT25) and later (i.e., 24PD) infection time points. During this time, the ant host shows a characteristic summiting behavior adaptive to the development and spread of *Ophiocordyceps* (Andersen et al., 2012, Will et al., 2022). Alpha diversity for both micro- and mycobiomes differed significantly between ants at the LM time point and ants from the control group, and the LT25 and 24PD timepoints, which may be an indication of a gradual change with infection progression. However, alpha diversity of the micro- and mycobiome did not differ between the other time points, suggesting that something uniquely different might be happening with gut microorganisms during manipulated summiting behavior. At the LM timepoint, the microbiome again showed a sudden drop in bacterial ASV richness while there was a significant increase in fungal ASV richness seen for the mycobiome. The only time point during which the gut micro- and mycobiota was not significantly different compared to the LM timepoint was death after manipulation (DM). For *Ophiocordyceps*-infected ants, death occurs within a mere few hours of live manipulation. In contrast, the other time points were separated from the LM time point by 24+ hours (24PD) to several days (LT25). As such, the relatively few hours difference between LM and DM time points may not have been enough time for the micro- and mycobiota to change significantly. However, the micro- and mycobiota of LM ants was significantly different from that of 24PD ants, while we did not observe significant differentiation between the DM and 24PD timepoints. This suggests that there is already a gradual change in ant gut micro- and mycobiota in the short amount of time between being live manipulated and eventually succumbing to the infection. While the beta diversity analysis corroborated these findings for the mycobiome, they did not do so entirely for the bacterial microbiome. This suggests again that the fungal composition of the gut appears to be more dynamic than the bacterial composition and that *Ophiocordyceps* infection is somehow significantly affecting that composition, especially during the manipulated summiting stage.

These different disease outcomes for the host gut micro- and mycobiota could be due to a direct effect of the pathogen on the host and its tissues. Alternatively, the differences we observed could be a result of changed behavior upon infection. Many social insects, including *Camponotus* ants, engage in trophallaxis – the social exchange of fluids between individuals. Such social fluids contain partially digested and regurgitated food materials as well as that individual's endocrine molecules that play a role in maintaining the social network and decision-making within an ant colony. Trophallaxis is also hypothesized to play a role in inoculating and maintaining gut microbiota among colony members (Meurville and LeBoeuf, 2021). Thus, a disconnect from the social network of the colony could have potential impacts on an individual's microbiome. Indeed, *Ophiocordyceps*-infected ants display diminished communication with nestmates (Trinh et al., 2021), which consequently might reduce trophallaxis. As such, they might be disconnected from their colony mates with the consequence that their regular gut microbiota is not maintained. While the period between infection, manipulation and eventual death spans several weeks in *Ophiocordyceps, Beauveria* infection runs a more rapid course of 5 days to a week. Diminished communication was not readily observed in *C. floridanus* infected with *Beauveria* (Trinh et al., 2021). However, even if reduced communication were to occur, the disruption might be too brief to have a measurable influence on the micro- and mycobiota. Therefore, a prolonged detachment from the social-fluid exchange due to asocial behavior might result in the microbiome dysbiosis seen in *Ophiocordyceps*-infected ants, which could explain the differences between the microbiomes of *Ophiocordyceps*- versus *Beauveria*-infected ants. Nevertheless, if this were the case, one might expect a more gradual change over time with significance compared to controls being found for all later stages of infection (LM, DM and 24PD) and not just the single live manipulation time point.

Of course, we cannot completely exclude that the non-significant results we obtained for *Beauveria* are caused by the nature of our samples and sample size. Additionally, the different time scales of the infections might have a role to play; the short infection period for *Beauveria* might not be enough to have a significant effect on the gut composition. The survival curve for the *Ophiocordyceps* infections (Fig. 1A) followed expected trends with most infected ants dying once manipulations began (21 DPI). The survival curve for *Beauveria* infections (Fig. 1B) also matched expected trends with a decline in survival starting between 3-5 DPI. However, the survival rate levelled out after 6 DPI with around 50% of the population still alive. This indicates that not all ants progressed through the infection the same way and that some were able to clear it. The sick (LS), dead (DS), and 24h post death (24PD) *Beauveria* timepoint samples are unaffected by this, as they were sampled based on a distinguishable sick behavior phenotype. However, the LT25 timepoint samples may be a mixture of infected and uninfected individuals as there was no clear way to distinguish infection status for those ants. Moreover, sample size was limited for each timepoint and might not have been large enough to reveal the full effects of fungal infection over time. Yet, the presence of dysbiosis in the guts of *Ophiocordyceps*-infected ants that displayed summiting behavior was clearly significantly different compared to most other infection time points, including healthy ants. In fact, looking at the NMDS plots (Fig. 3C and D), the LM samples cluster together while the other biological groups seem to show a more dispersed level of variation. As such, we consider the finding that *Ophiocordyceps* infection affects the gut micro- and mycobiome of ants during manipulated summiting to be evident.

The biological relevance of a distinctly altered gut microbiome composition during manipulated summiting behavior remains a topic of speculation. One possibility could be that the ant gut microbiome affects host behavior and may be involved, to some degree, in the expression of stereotypically altered behaviors during *Ophiocordyceps* infection. There is mounting evidence from human research that gut dysbiosis is associated with an increased risk of Alzheimer's, depression, and other behavioral pathologies (Krautkramer et al., 2021). Some studies suggest that this may occur via microbial metabolites secreted in the gut interacting with neurological receptors and G protein coupled receptors (GPCRs) (Aleti et al., 2023, Cohen et al., 2017). Transcriptomic analyses of *Ophiocordyceps*-infected ants that displayed manipulated summiting behavior showed enrichment for GPCRs among the genes that were downregulated during this infection stage as compared to healthy individuals (Will et al., 2020). These GPCRs were annotated to have functions involved in the perception of light and smell, and the binding of biogenic amines such as dopamine. Most of the pathogen-related behavioral changes modulated by these GPCRs are likely due to the predicted binding of *Ophiocordyceps* secreted proteins that are upregulated during manipulated summiting (Will et al., 2023). However, other GPCRs might be interacting with (altered levels of) microbial peptides secreted by the microbiome. Additionally, there is increasing evidence that gut microbiomes aid in insect host immune defense and microbiome homeostasis is thought to be an important factor in mediating the immune system (Boucias et al., 2018, Raymann and Moran, 2018). For instance, recent research into honeybee core microbiomes showed that dysbiosis was linked to higher susceptibility to pathogen invasion and consequently higher mortality rates (Raymann and Moran, 2018). Considering that a hemibiotroph-like pathogen such as *Ophiocordyceps* needs to ensure prolonged colonization and successful manipulation of the ant without causing significant physical damage, this fungus could perhaps be affecting the microbiome such that the host immune response is effectively lowered. This hypothesis would be in line with metabolomics data that suggest a lowered immune response in the ant paired with the presence of neuroprotectants (Will et al., 2023, Loreto and Hughes, 2019). However, one would expect this strategy to be needed during the earlier time points in infection as well, where we did not detect any significant micro- nor mycobiome changes. Taken together, the potential pathogen-adaptive function of gut dysbiosis during *Ophiocordyceps* infection, if any, remains to be discovered. Nevertheless, both the hypotheses we propose here could provide an explanation as to why we do detect this dysbiosis of the host gut in infections with the hemibiotroph-like, behavior-manipulating specialist *Ophiocordyceps* and not in those with the necrotroph-like, non-manipulating generalist *Beauveria*.

## 5 Conclusions

To our knowledge, all previous research done on the gut microbiota of ants has focused on their bacterial microbiome, making our study the first to investigate the fungal taxa present within the ant gut, the mycobiome. Our data show that, while the bacterial microbiome is often dominated by one species, the fungal mycobiome is highly diverse with more evenness between taxa. Moreover, our data suggest that the fungal taxonomic composition of the ant gut is more dynamic, as community changes were more prominently detected in the fungal components of the gut. These results support the notion that the fungal portion of the microbiome likely plays a more prominent role in insect functioning than previously credited and should be studied more intensely. Moreover, we provide the first evidence that the gut microbiome of *C. floridanus*, and with that perhaps also other insects, appears to be impacted differently by infecting entomopathogens with different lifestyles. Of course, it needs to be noted here that we only compared two fungal pathogens to arrive at this hypothesis, which could use strengthening of future studies that would include more species. More specifically, our data suggest that behavior-manipulating specialists like *O. camponoti-floridani* might affect the host microbiome in a timed manner that aligns with pathogen-adaptive host behavioral phenotypes. As such, considering the role that microbiota might play in host behavior, we speculate that part of the behavioral manipulation might be the result of dysbiosis of the gut microbiota. Research in which specific modulation of gut taxa is linked to host behavior and infection status will be needed to begin to unravel these potential intersections. This will improve our understanding of the gut-brain axis and provide knowledge that could improve the effective use of fungal pathogens in the biocontrol of insect pests.

## **CRediT authorship contribution statement**

**Sophia Vermeulen:** Data curation, Formal Analysis, Investigation, Methodology, Writing – original draft. **Anna M Forsman:** Formal Analysis, Methodology, Supervision, Writing – review and editing. **Charissa de Bekker:** Conceptualization, Formal Analysis, Funding acquisition, Methodology, Supervision, Writing – review and editing.

## Declaration of competing interest

The authors declare that they have no known competing financial interests or personal relationships that could have appeared to influence the work reported in this paper.

## Data Availability

All sequence data generated in this study have been deposited in the NCBI Sequence Read Archive under BioProject ID PRJNA1129269.

## Appendix Supplementary materials

Image, application 1

Image, application 2

Image, application 3

Image, application 4
