# Supplementary material for: Consequences of “zombie-making” and generalist fungal pathogens on carpenter ant microbiota
Source: Curr Res Insect Sci. 2024 Nov 30;7:100102. doi: 10.1016/j.cris.2024.100102 (PMC11665668; doi:10.1016/j.cris.2024.100102)
Supplement: Supplementary file 3 [file mmc3.docx]

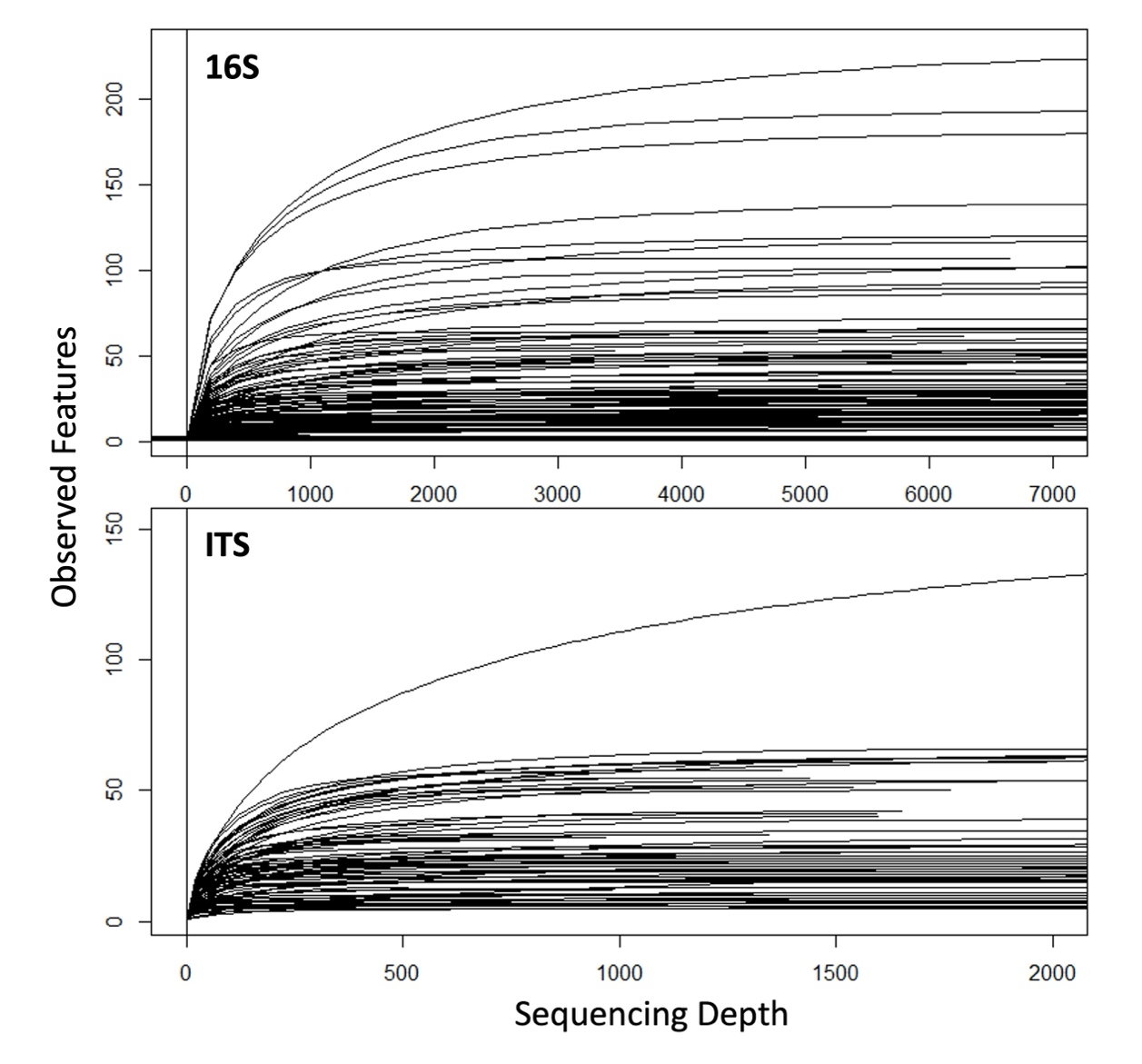


***Supplementary Figure 1: Rarefaction Curves for 16S (top) and ITS reads (bottom).*** *Rarefaction curves indicate sufficient species coverage at a sequence sampling depth of 4000 for 16S libraries, and 500 for ITS libraries. Observed features represent the number of different ASV’s identified.*

|  |  | 16S libraries | | | ITS libraries | | |
| --- | --- | --- | --- | --- | --- | --- | --- |
|  |  | *Ophiocordyceps* | *Beauveria* | Control | *Ophiocordyceps* | *Beauveria* | Control |
| **Total** | | **33** | **37** | **24** | **38** | **23** | **20** |
| LT25 | | 8 | 10 |  | 8 | 7 |  |
| LM/LS | | 11 | 10 |  | 13 | 5 |  |
| DM/DS | | 9 | 9 |  | 8 | 6 |  |
| 24PD | | 5 | 8 |  | 9 | 5 |  |

***Supplementary Table 1: Samples remaining after read filtering and rarefaction.*** *Ten samples were collected at each timepoint, except for Ophiocordyceps ‘live manipulated’ where 13 samples were collected and sequenced. Control samples were pooled for both experiments, from which we randomly selected 24 samples for processing.*


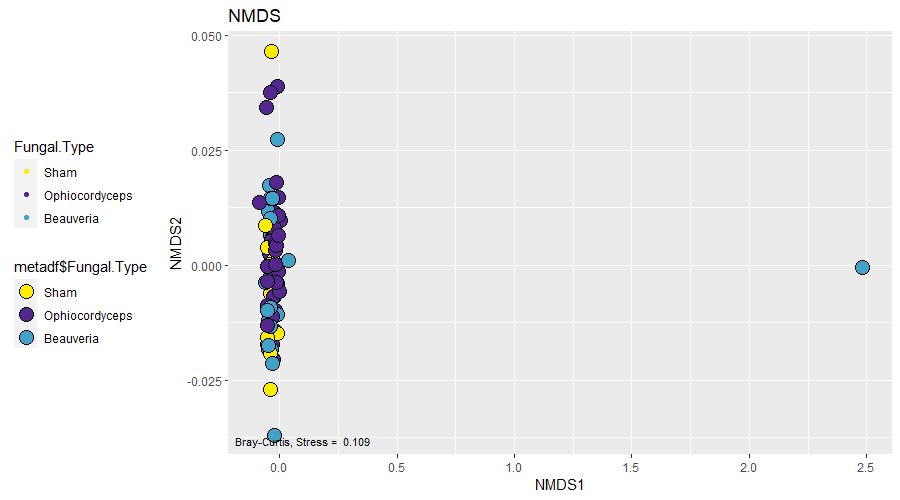


***Supplementary Figure 2: ITS NMDS plot with outlier Beauveria sample included.*** *Non-metric multidimensional scaling (NMDS) plot using a Bray Curtis distance matrix (stress = 0.109) of the gut mycobiome (ITS) for different treatment groups. The points represent microbiomes of individual samples, and the colors indicate which treatment groups the samples belong to (yellow = control, purple = Ophiocordyceps, blue = Beauveria). The blue dot at the far right of the graph represents the outlier from the Beauveria treatment group that was removed for analysis. After applying read filtering, and removal of Ophiocordyceps and Beauveria reads, this sample was only left with unidentified reads and reads identified as Malassezia, a likely contaminant.*

*
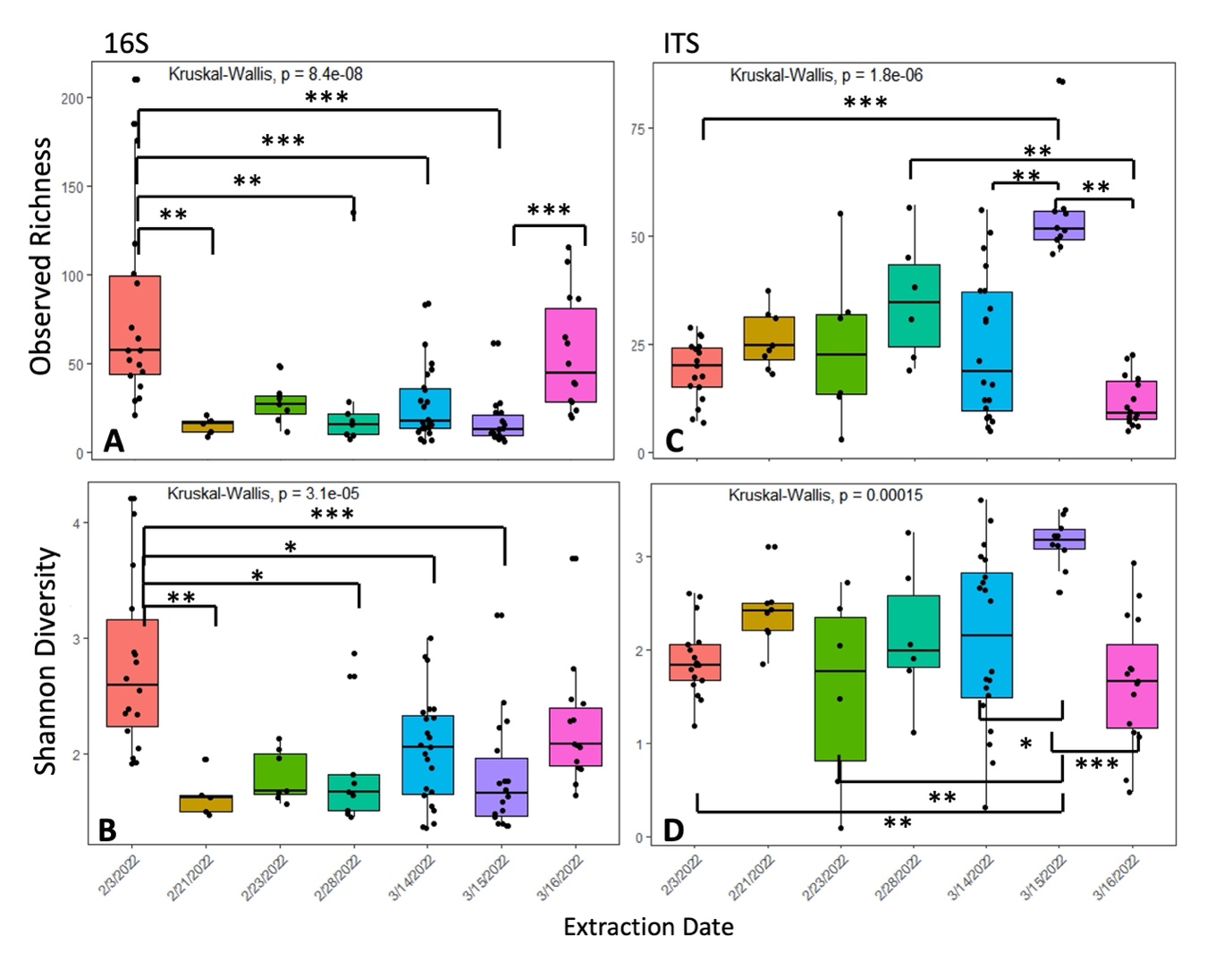
*

***Supplementary Figure 3: Alpha diversity plots of extraction batches for 16S and ITS libraries.*** *A) Observed richness in the bacterial microbiome (16S) of extraction batches containing random samples. B) Shannon diversity index of the bacterial microbiome of extraction batches. C) Observed richness for the mycobiome (ITS) of extraction batches. D) Shannon diversity index for the mycobiome of extraction batches. P-value significance between extraction batches containing random samples from this study: * <0.05, ** < 0.01, *** <0.001.*

*
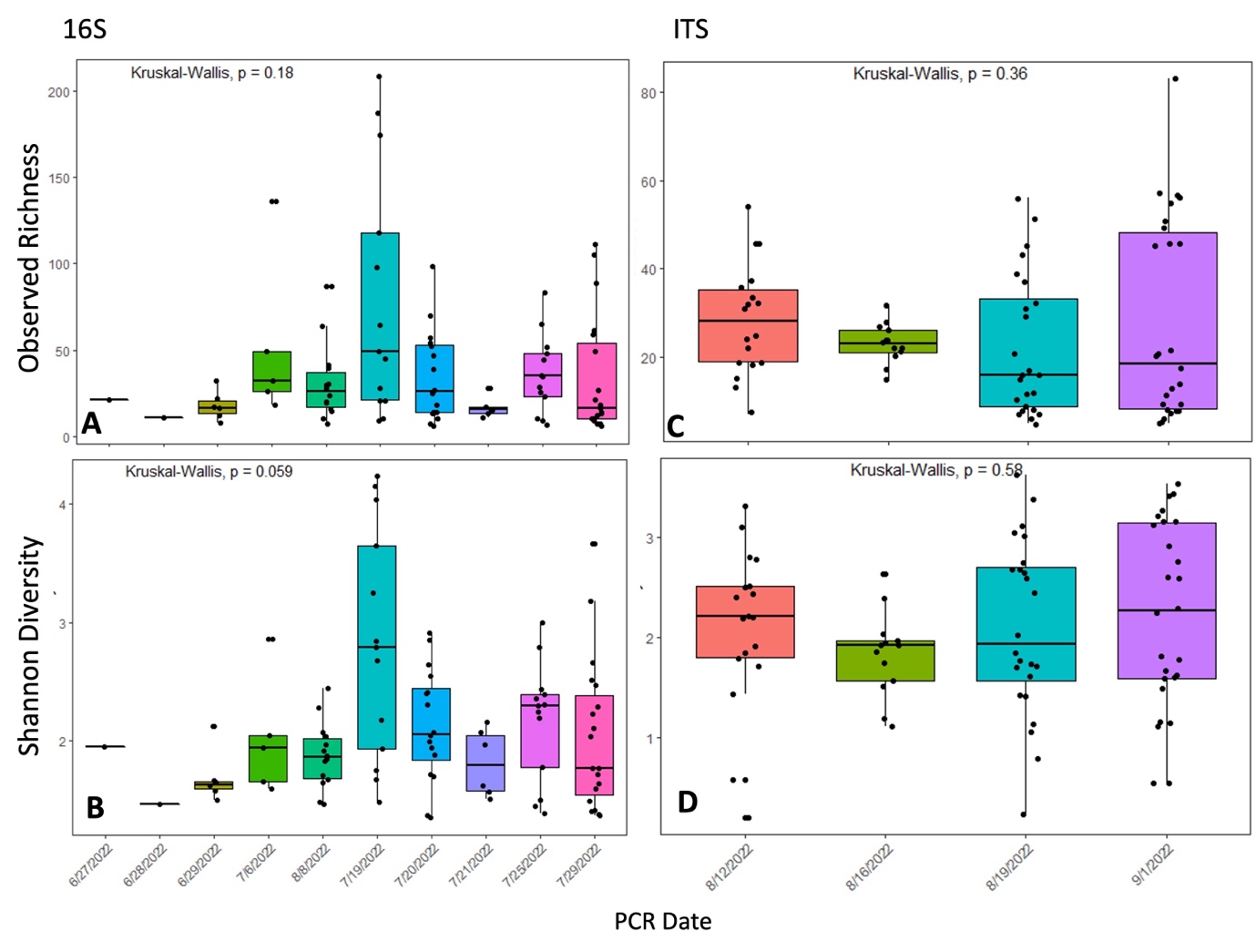
*

***Supplementary Figure 4: Alpha diversity plots of PCR batches for 16S and ITS libraries.*** *A) Observed richness in the bacterial microbiome (16S) of PCR batches containing random samples from this study. B) Shannon diversity index of the bacterial microbiome of PCR batches. C) Observed richness for the mycobiome (ITS) of PCR batches. D) Shannon diversity index for the mycobiome of PCR batches. No significant differences were seen between PCR batches containing random samples from this study.*
