# Supplementary material for: Consequences of “zombie-making” and generalist fungal pathogens on carpenter ant microbiota
Source: Curr Res Insect Sci. 2024 Nov 30;7:100102. doi: 10.1016/j.cris.2024.100102 (PMC11665668; doi:10.1016/j.cris.2024.100102)
Supplement: Supplementary file 4 [file mmc4.docx]

|  | **Observed Richness** | | **Shannon Diversity** |  |
| --- | --- | --- | --- | --- |
| Pairwise comparison | 16S | ITS | 16S | ITS |
| *Ophiocordyceps - Beauveria* | **0.003** | **0.006** | **0.0003** | **0.01** |
| *Ophiocordyceps* - Control | 0.123 | 0.69 | 0.63 | **0.01** |
| *Beauveria* - Control | 1 | 0.34 | 0.073 | 1 |

***Supplementary Table 2: Dunn test results for alpha diversity comparisons between treatments.*** *P-values have been adjusted with the Bonferroni correction for multiple comparisons. P-values in bold font indicate significance (p < 0.05).*

**16S**

| Pairwise comparison | Df | SumOfSqs | F.Model | R2 | P-value |
| --- | --- | --- | --- | --- | --- |
| *Ophiocordyceps - Beauveria* | 1 | 0.368 | 3.04 | 0.043 | **0.033** |
| *Ophiocordyceps -* Control | 1 | 0.098 | 1.18 | 0.021 | 0.882 |
| *Beauveria –* Control | 1 | 0.320 | 2.47 | 0.040 | 0.069 |
| **ITS** |  |  |  |  |  |
| Pairwise comparison | Df | SumOfSqs | F.Model | R2 | P-value |
| *Ophiocordyceps – Beauveria* | 1 | 1.6 | 4.61 | 0.0725 | **0.003** |
| *Ophiocordyceps* - Control | 1 | 1.58 | 4.84 | 0.0795 | **0.003** |
| *Beauveria -* Control | 1 | 0.34 | 1.03 | 0.0245 | 1 |

***Supplementary Table 3: Pairwise PERMANOVA results for the beta diversity comparisons between treatments.*** *P-values have been adjusted with the Bonferroni correction for multiple comparisons. P-values in bold indicate significance (p<0.05).*

| ***Ophiocordyceps*** | **Observed Richness** | | **Shannon Diversity** | |
| --- | --- | --- | --- | --- |
| Pairwise comparison | 16S | ITS | 16S | ITS |
| Control– LT25 | 1 | 1 | 1 | 1 |
| Control– LM | **0.005** | **0.014** | 0.11 | **<0.0001** |
| Control– DM | 0.34 | 0.92 | 0.76 | 0.60 |
| Control– 24PD | 1 | 1 | 1 | 1 |
| LT25 – LM | **0.022** | **0.006** | 0.14 | **0.012** |
| LT25 – DM | 0.43 | 0.27 | 0.63 | 1 |
| LT25 – 24PD | 1 | 1 | 1 | 1 |
| LM– DM | 1 | 1 | 1 | 0.53 |
| LM– 24PD | **0.034** | **0.048** | 0.068 | **0.034** |
| DM– 24PD | 0.41 | 1 | 0.28 | 1 |

***Supplementary Table 4: Dunn test results for alpha diversity comparisons between Ophiocordyceps infection timepoints.*** *P-values have been adjusted with the Bonferroni correction for multiple comparisons. P-values in bold font indicate significance (p < 0.05).*

| ***Ophiocordyceps* 16S** |  |  |  |  |  |
| --- | --- | --- | --- | --- | --- |
| Pairwise comparisons | Df | SumsOfSqs | F.Model | R2 | P-value |
| 24PD vs Control | 1 | 0.385 | 3.38 | 0.111 | 0.23 |
| 24PD vs DM | 1 | 0.398 | 3.37 | 0.219 | 0.07 |
| 24PD vs LM | 2 | 0.464 | 2.38 | 0.268 | **0.04** |
| 24PD vs LT25 | 1 | 0.263 | 1.98 | 0.152 | 0.66 |
| Control vs DM | 1 | 0.076 | 1.01 | 0.032 | 1 |
| Control vs LM | 1 | 0.126 | 1.89 | 0.054 | 0.8 |
| Control vs LT25 | 2 | 0.043 | 0.26 | 0.018 | 1 |
| DM vs LM | 1 | 0.016 | 0.52 | 0.028 | 1 |
| DM vs LT25 | 1 | 0.045 | 0.92 | 0.058 | 1 |
| LM vs LT25 | 1 | 0.083 | 2.42 | 0.125 | 0.33 |
| ***Ophiocordyceps* ITS** |  |  |  |  |  |
| Pairwise comparisons | Df | SumsOfSqs | F.Model | R2 | P-value |
| 24PD vs Control | 1 | 0.96 | 3.16 | 0.105 | **0.01** |
| 24PD vs DM | 1 | 0.34 | 1.01 | 0.063 | 1 |
| 24PD vs LM | 2 | 1.13 | 1.99 | 0.174 | **0.01** |
| 24PD vs LT25 | 1 | 0.57 | 1.64 | 0.098 | 0.27 |
| Control vs DM | 1 | 0.87 | 2.81 | 0.098 | 0.06 |
| Control vs LM | 1 | 2.27 | 8.35 | 0.212 | **0.01** |
| Control vs LT25 | 2 | 0.31 | 0.47 | 0.036 | 1 |
| DM vs LM | 1 | 0.43 | 1.57 | 0.076 | 0.86 |
| DM vs LT25 | 1 | 0.52 | 1.45 | 0.094 | 1 |
| LM vs LT25 | 1 | 1.27 | 4.46 | 0.190 | **0.02** |

***Supplementary Table 5: Pairwise PERMANOVA results for the beta diversity comparisons between Ophiocordyceps infection timepoints****. P-values have been adjusted with the Bonferroni correction for multiple comparisons. P-values in bold indicate significance (p<0.05).*

| ***Beauveria*** | **Observed Richness** | | **Shannon Diversity** | |
| --- | --- | --- | --- | --- |
| Pairwise comparison | 16S | ITS | 16S | ITS |
| Control – LT25 | 1 | **0.034** | 1 | 1 |
| Control – LS | 1 | 1 | 1 | 1 |
| Control – DS | 1 | 1 | 0.24 | 1 |
| Control – 24PD | 1 | 1 | 1 | 1 |
| LT25 – LS | 1 | 0.40 | 1 | 1 |
| LT25 – DS | 1 | 0.63 | 1 | 1 |
| LT25 – 24PD | 1 | 1 | 1 | 1 |
| LS – DS | 1 | 1 | 1 | 1 |
| LS – 24PD | 1 | 1 | 1 | 1 |
| DS – 24PD | 1 | 1 | 1 | 1 |

***Supplementary Table 6: Dunn test results for alpha diversity comparisons between Beauveria infection timepoints.*** *P-values have been adjusted with the Bonferroni correction for multiple comparisons. P-values in bold font indicate significance (p < 0.05).*

| ***Beauveria* 16S** |  |  |  |  |  |
| --- | --- | --- | --- | --- | --- |
| Pairwise comparisons | Df | SumsOfSqs | F.Model | R2 | P-value |
| Control vs 24PD | 1 | 0.859 | 6.53 | 0.179 | **0.01** |
| Control vs LT25 | 2 | 0.047 | 0.28 | 0.017 | 1 |
| Control vs DS | 1 | 0.211 | 1.94 | 0.059 | 1 |
| Control vs LS | 1 | 0.125 | 1.41 | 0.042 | 1 |
| 24PD vs LT25 | 1 | 0.597 | 3.68 | 0.187 | **0.04** |
| 24PD vs DS | 1 | 0.266 | 1.21 | 0.074 | 1 |
| 24PD vs LS | 2 | 0.552 | 1.50 | 0.167 | 0.1 |
| LT25 vs DS | 1 | 0.130 | 1.09 | 0.060 | 1 |
| LT25 vs LS | 1 | 0.077 | 0.94 | 0.050 | 1 |
| DS vs LS | 1 | 0.148 | 1.14 | 0.063 | 1 |
| ***Beauveria* ITS** |  |  |  |  |  |
| Pairwise comparisons | Df | SumsOfSqs | F.Model | R2 | P-value |
| Control vs 24PD | 1 | 0.321 | 1.027 | 0.043 | 1 |
| Control vs LT25 | 2 | 0.284 | 0.446 | 0.036 | 1 |
| Control vs LS | 1 | 0.284 | 0.896 | 0.037 | 1 |
| Control vs DS | 1 | 0.370 | 1.195 | 0.047 | 1 |
| 24PD vs LT25 | 1 | 0.274 | 0.771 | 0.072 | 1 |
| 24PD vs LS | 1 | 0.286 | 0.714 | 0.082 | 1 |
| 24PD vs DS | 1 | 0.309 | 0.833 | 0.085 | 1 |
| LT25 vs LS | 1 | 0.285 | 0.782 | 0.073 | 1 |
| LT25 vs DS | 1 | 0.332 | 0.967 | 0.081 | 1 |
| LS vs DS | 1 | 0.290 | 0.759 | 0.078 | 1 |

***Supplementary Table 7: Pairwise PERMANOVA results for the beta diversity comparisons between Beauveria infection timepoints.*** *P-values have been adjusted with the Bonferroni correction for multiple comparisons. P-values in bold indicate significance (p<0.05).*
